# Characteristics and Patterns of Retention in Hypertension Care in Primary Care Settings From the Hypertension Treatment in Nigeria Program

**DOI:** 10.1001/jamanetworkopen.2022.30025

**Published:** 2022-09-06

**Authors:** Jiancheng Ye, Ikechukwu A. Orji, Abigail S. Baldridge, Tunde M. Ojo, Grace Shedul, Eugenia N. Ugwuneji, Nonye B. Egenti, Kasarachi Aluka-Omitiran, Rosemary C. B. Okoli, Helen Eze, Ada Nwankwo, Lisa R. Hirschhorn, Aashima Chopra, Boni M. Ale, Gabriel L. Shedul, Priya Tripathi, Namratha R. Kandula, Mark D. Huffman, Dike B. Ojji

**Affiliations:** 1 Feinberg School of Medicine, Northwestern University, Chicago, Illinois; 2 Cardiovascular Research Unit, University of Abuja Teaching Hospital, Gwagwalada, Abuja, Nigeria; 3 University of Nigeria, Nsukka, Nigeria; 4 Holo Healthcare, Nairobi, Kenya; 5 The George Institute for Global Health, Sydney, New South Wales, Australia; 6 Washington University in St Louis, St Louis, Missouri; 7 University of Abuja, Abuja, Nigeria

## Abstract

**Background:**

More than 1.2 billion adults worldwide have hypertension. High retention in clinical care is essential for long-term management of hypertension, but 1-year retention rates are less than 50% in many resource-limited settings.

**Objective:**

To evaluate short-term retention rates and associated factors among patients with hypertension in primary health care centers in the Federal Capital Territory of Nigeria.

**Design, Setting, and Participants:**

In this cohort study, data were collected by trained study staff from adults aged 18 years or older at 60 public, primary health care centers in Nigeria between January 2020 and July 2021 as part of the Hypertension Treatment in Nigeria (HTN) Program. Patients with hypertension were registered.

**Exposures:**

Follow-up visit for hypertension care within 37 days of the registration visit.

**Main Outcomes and Measures:**

The main outcome was the 3-month rolling average 37-day retention rate in hypertension care, calculated by dividing the number of patients who had a follow-up visit within 37 days of their first (ie, registration) visit in the program by the total number of registered patients with hypertension during multiple consecutive 3-month periods. Interrupted time series analyses evaluated trends in retention rates before and after the intervention phase of the HTN Program. Mixed-effects, multivariable regression models evaluated associations between patient-, site-, and area council–level factors, hypertension treatment and control status, and 37-day retention rate.

**Results:**

In total, 10 686 patients (68.3% female; mean [SD] age, 48.8 [12.7] years) were included in the analysis. During the study period, the 3-month rolling average 37-day retention rate was 41% (95% CI, 37%-46%), with wide variability among sites. The retention rate was higher among patients who were older (adjusted odds ratio [aOR], 1.01 per year; 95% CI, 1.01-1.02 per year), were female (aOR, 1.11; 95% CI, 1.01-1.23), had a higher body mass index (aOR, 1.01; 95% CI, 1.00-1.02), were in the Kuje vs the Abaji area council (aOR, 2.25; 95% CI, 1.25-4.04), received hypertension treatment at the registration visit (aOR, 1.27; 95% CI, 1.07-1.50), and were registered during the postintervention period (aOR, 1.16; 95% CI, 1.06-1.26).

**Conclusions and Relevance:**

The findings suggest that retention in hypertension care is suboptimal in primary health care centers in Nigeria, although large variability among sites was found. Potentially modifiable and nonmodifiable factors associated with retention were identified and may inform multilevel, contextualized implementation strategies to improve retention.

## Key Points

**Question:**

What are characteristics and patterns of retention in hypertension care in primary care settings in Nigeria?

**Findings:**

In this cohort study of 10 686 patients with hypertension at 60 primary health care centers, the 3-month rolling average 37-day retention rate was 41%, with wide variability among centers. The retention rate was higher among patients who were older, were female, had a higher body mass index, lived in the Kuje area council, received hypertension treatment at the registration visit, and were registered during the postintervention period.

**Meaning:**

The findings suggest that retention in hypertension care is suboptimal in primary health care centers in Nigeria, although variability among sites was found.

## Teaser

This cohort study examines short-term retention rates in hypertension care and factors associated with retention among adult patients with hypertension in public primary health care centers in Nigeria.

## Introduction

Elevated blood pressure is a leading modifiable factor associated with cardiovascular disease morbidity and mortality in high-income countries, such as the US, and in low- and middle-income countries.^1,2^ Approximately 1.28 billion adults aged 30 to 79 years worldwide have hypertension, and two-thirds live in low- and middle-income countries.^3^ Nigeria is the most populous country in Africa and has an estimated hypertension prevalence of 38%, with suboptimal hypertension awareness (60%), treatment (34%), and control (12%) rates.^4^ Nigeria has set a national goal of reducing the risk of premature mortality from noncommunicable diseases by one-third by 2030.^5^ To reduce the risk of hypertension-related diseases and to improve healthy life expectancy, hypertension care in Nigeria is transitioning from secondary and tertiary care centers to primary health care centers (PHCs) to improve the reach, effectiveness, implementation, and maintenance of hypertensive services, as recommended by the World Health Organization (WHO) healthy lifestyle counseling, evidence-based treatment protocols, access to essential medicines and technology, risk-based cardiovascular disease management, team-based care, and systems for monitoring (HEARTS) technical package.^6,7^ This multilevel intervention includes (1) a standard treatment protocol (national policy level), (2) patient registration and empanelment (health system level), (3) encouragement of fixed-dose combination therapy (health system level), (4) incentivized team-based care (health care worker level), and (5) home blood pressure monitoring and health coaching (patient level). The Hypertension Treatment in Nigeria (HTN) Program was created to implement and evaluate a culturally and contextually adapted, large-scale, evidence-based implementation package to improve the cascade of hypertension care in public PHCs in the Federal Capital Territory of Nigeria.

Retention to hypertension care is defined as patients’ regular engagement with medical care at a health care facility. High retention is essential for long-term management of hypertension and programmatic maintenance, but 1-year retention rates are less than 50% in many resource-limited settings.^8^ Optimizing retention in care is a central objective of the HTN Program because longitudinal, patient-centered care is essential for sustained hypertension control. Primary care services may be limited in scope, resources, and staff training to achieve high retention rates, and the COVID-19 pandemic has further threatened the hypertension care cascade from diagnosis to treatment and subsequent control.^9^ Despite the importance of retention in the hypertension care cascade, few studies have comprehensively described the characteristics and patterns of retention in hypertension care in primary care settings.^10,11,12^

This study aimed to describe the characteristics and patterns of short-term retention (follow-up ≤37 days to match international and national guidelines)^13,14^ in hypertension care in public PHCs in the Federal Capital Territory of Nigeria that are part of the HTN Program. We also sought to identify potentially modifiable factors associated with retention that would be the targets for multilevel, contextualized implementation strategies to improve patient adherence to hypertension care.

## Methods

### Ethics Review and Informed Consent

The protocol for this cohort study was reviewed and approved by the ethics boards of the University of Abuja Teaching Hospital, Federal Capital Territory of Nigeria, and Northwestern University, as well as the HTN Program’s data and safety monitoring board. Patient informed consent was waived based on the Common Rule. This study followed the Strengthening the Reporting of Observational Studies in Epidemiology (STROBE) reporting guideline.^15^

### Study Design and Population

This study was embedded within the HTN Program, which is a prospective, longitudinal, type-2 hybrid implementation-effectiveness research study. The HTN Program aims to evaluate the implementation and effectiveness of a multilevel, evidence-based implementation package using an interrupted time series design.^16^ The research team conducted a service availability and readiness assessment in 2019, and a multistage random sampling frame was used to select eligible PHCs based on geographic representation and population size, with additional sites selected after consultation with the Nigerian Federal Ministry of Health and the Federal Capital Territory’s Primary Health Care Board. Sixty PHCs were invited to participate in the HTN Program, and all agreed to participate. Details about the HTN Program are reported elsewhere.^17,18^

The current study included 19 months of data from the HTN Program, among which the first 11 months (January to November 2020) represent the preintervention, or baseline control, period and the remaining 8 months (December 2020 to July 2021) represent the postintervention period. Follow-up data from August to October 2021 were also included to evaluate retention and follow-up patterns within 3 months after baseline registration from January 2020 to July 2021. The multilevel implementation package was based on the Kaiser Permanente Northern California model^19^ and the WHO HEARTS technical package.^7^ Frontline health care staff are compensated for registration of patients through monthly stipends of at least 10 000 naira (USD 24) each. The home blood pressure monitoring and health coaching intervention will be implemented in the third quarter of 2022. Starting in January 2021, blood pressure–lowering medications were made freely available to patients in 30-day supplies as part of the development of a drug revolving fund for the sustainment of a reliable supply of quality medications.

Despite the recognized clinical and programmatic importance of retention, there is no recognized standard measure for retention in care. Measuring retention is complex because it includes multiple visits, scheduled at varying time intervals, and occurring across time. There are many ways to define and operationalize retention in care, and studies have used a wide range of approaches.^20^ This study focused on the retention between the registration visit and the first follow-up visit within 37 days based on the WHO and Nigeria national hypertension guidelines, both of which recommend 1-month follow-up.^13,14^ We chose 37 days rather than 1 month or 30 days as the primary time point to measure retention for the following reasons: (1) patients were typically prescribed 30-day treatment regimens; (2) documentation of patient registration and medical records was paper based, and the record information officers needed time to manually enter the data into REDCap; (3) the research team conducted biweekly data status and quality reports checks to identify missing and low-quality data, and the respective site-level record information officers verified or corrected data based on these reports, which may have had time lags or delays;^21^ and (4) although practitioners informed each patient to follow up in 1 month or 30 days, the current study used a modestly longer window to avoid misclassifying patients who followed up just beyond the recommended period. Although longer (eg, 90-day) prescriptions have been recommended to improve hypertension control,^22^ the out-of-pocket costs for longer treatment regimens make this infeasible for many Nigerians.^23^

We developed monthly, automated audit and feedback reports (eFigure 1 in the Supplement) to assess study quality and performance and a real-time registry dashboard for patient follow-up tracking; these were designed to improve retention through patient empanelment. The audit and feedback reports provided information on a 3-month rolling basis, including (1) the number of newly registered patients by month, (2) the number of days since the last visit among all registered patients, (3) the proportion of patients who were treated, (4) the proportion of patients for whom blood pressure was controlled, (5) patients who should be contacted to return to the PHC, and (6) patients who should be considered for treatment with a first-line blood pressure–lowering drug at their next visit or for an additional medication if already taking a medication. The automated audit and feedback reports assisted the study team in performing quarterly site-level monitoring and supportive supervision visits.

### Study Population

Sites were instructed to document patient visits and to measure blood pressure twice among all adults (age ≥18 years) who presented to the participating PHC using a standard approach following centralized training and periodic retraining at the University of Abuja Teaching Hospital. Patients with hypertension were registered into a longitudinal hypertension registry regardless of the baseline or intervention period. Consecutive adults with hypertension, defined as (1) a history of hypertension, (2) persistently elevated systolic blood pressure of 140 mm Hg or above, (3) persistently elevated diastolic blood pressure of 90 mm Hg or above, and/or (4) use of blood pressure–lowering medications, were registered at their respective PHC. Persistently elevated blood pressure was defined based on 2 or more measurements taken at least 1 week apart at respective health centers based on the 2021 WHO hypertension guideline.^22^ Patients who had not been previously diagnosed with hypertension and presented with systolic blood pressure between 140 and 150 mm Hg would have their blood pressure rechecked in 3 to 7 days for confirmation of persistently elevated blood pressure. For patients with systolic blood pressure higher than 150 mm Hg, their blood pressure would be rechecked on the same day within 1 to 3 hours.

After diagnosis of hypertension or a visit to the PHC during the registration period, participant data were captured on a participant’s treatment card by a trained health care worker at the PHC. The participant was administered a hypertension treatment card, including a unique hypertension program identifier, blood pressure, heart rate, body weight, and medications. Participants were instructed to bring this card to subsequent visits to track their blood pressure over time. Participant treatment cards were also retained by the PHCs to capture baseline and longitudinal participant information including demographics, contact information, medical history, blood pressure measurements, laboratory measures, hypertension medications, and adverse events.^24^

At subsequent patient visits, diagnosis and treatment cards and paper case report forms were updated with blood pressure measurements; changes in blood pressure–lowering medications, if any; and available laboratory data. The research team performed centralized monitoring through data and status quality reports and REDCap, reporting features to identify missing and low-quality data, including outliers and data entry errors. The research team reviewed sites’ reports with the PHCs’ respective record information officers to verify or correct data based on source document review.

### Statistical Analysis

Deidentified descriptive data are reported as means (SDs) and medians (IQRs) for continuous variables if data were skewed and as proportions (95% CIs) for categorical variables. Data were analyzed overall and by key subgroups, including by sex, PHC, area council, and calendar month. The retention, or follow-up, rate was calculated by dividing the number of patients who had a follow-up visit within a prespecified number of days (ie, 37) after their registration visit by the total number of registered patients with hypertension. We also assessed retention rates for other time points (ie, 3, 6, and 12 months), but these longer periods are beyond the primary focus of this study. The 3-month rolling average 37-day retention rate in hypertension care was calculated by dividing the number of patients who had a follow-up visit within 37 days of their first (ie, registration) visit by the total number of registered patients with hypertension during multiple consecutive 3-month periods. Other periods were included as sensitivity analyses.

All analyses were completed using a complete case approach. Interrupted time series analyses were performed to evaluate linear and nonlinear trends in retention rates before and after the intervention phase of the HTN Program. We used the root-mean-square error to evaluate the model fit.^25^ The intraclass correlation coefficient was used to measure the variability of the retention rates among sites. We also created hierarchical mixed-effects, multivariable logistic regression models to evaluate associations between patient demographics, comorbidities, site, area council–level factors, hypertension treatment and control, and site-level factors and 37-day retention rates. Unadjusted and adjusted models are reported. Models were adjusted for age, sex, body mass index (calculated as weight in kilograms divided by height in meters squared), alcohol use, fixed-dose combination therapy, and educational level. Random effects modeling was used to account for within-site clustering. SAS, version 9.4 (SAS Institute) and R, version 4.0.5 (R Project for Statistical Computing) were used for statistical analyses. A 2-sided *P* < .05 was used to define statistical significance, and no adjustments were made for multiple comparisons.

## Results

From January 2020 to July 2021, 11 078 unique patients were recruited and registered from 60 PHCs in 6 area councils. After excluding individuals with missing or erroneous data on age, sex, or blood pressure or who did not meet diagnostic criteria for hypertension (3.5%), 10 686 patients were included in the current analysis (eFigure 2 in the Supplement); among these, there were 36 925 unique visits (eTable 1 in the Supplement).

Table 1 reports patients’ demographics and clinical characteristics overall and by sex. The mean (SD) age was 48.8 (12.7) years; 68.3% of patients were female, 31.7% were male, and 26.6% had never attended school. Most patients (53.7%) had a history of hypertension, but self-reported history of comorbid disease, including diabetes (5.2%), heart failure (0.3%), chronic kidney disease (0.2%), stroke (0.8%), and heart attack (0.2%), was uncommon. The hypertension treatment rate at the start of the baseline visit was 52.0%. Current smoking was reported in 0.9% of patients, with a higher smoking rate among males compared with females (2.0% vs 0.3%; *P* < .001). The mean (SD) body mass index was higher among females compared with males (28.0 [6.3] vs 26.4 [4.9]; *P* < .001). The mean (SD) second systolic blood pressure measured was higher among males compared with females (153.5 [20.2] mm Hg vs 151.3 [20.9] mm Hg; *P* < .001), but mean diastolic blood pressure levels were similar between the groups.

**Table 1.** Patients’ Sociodemographic and Clinical Characteristics and Blood Pressure Overall and by Sex

Variables	Patients^a^			*P* value
	Total (n = 10 686)	Males (n = 3390)	Females (n = 7296)	
Age, mean (SD), y	48.8 (12.7)	52.2 (12.0)	47.2 (12.7)	<.001
Pregnant	NA	NA	113 (1.5)	NE
BMI, mean (SD)	27.5 (6.0)	26.4 (4.9)	28.0 (6.3)	<.001
Educational level				
Never attended school	2845 (26.6)	572 (16.9)	2273 (31.2)	<.001
Primary school	1991 (18.6)	565 (16.7)	1426 (19.5)	
Secondary school	2579 (24.1)	773 (22.8)	1806 (24.8)	
High school or more	3250 (30.5)	1472 (43.4)	1778 (24.4)	
Medical history				
Hypertension	5733 (53.7)	1773 (52.3)	3960 (54.3)	.046
Diabetes	559 (5.2)	175 (5.2)	384 (5.3)	.79
Heart failure	34 (0.3)	11 (0.3)	23 (0.3)	.43
Chronic kidney disease	16 (0.1)	6 (0.2)	10 (0.1)	.99
Stroke	84 (0.8)	48 (1.4)	36 (0.5)	<.001
Heart attack	25 (0.2)	12 (0.4)	13 (0.2)	.21
Smoking				
Current	92 (0.9)	69 (2.0)	23 (0.3)	<.001
Former	91 (0.9)	77 (2.3)	14 (0.2)	<.001
Alcohol use				
Current	385 (3.6)	271 (8.0)	114 (1.6)	<.001
Former	191 (1.8)	125 (3.7)	66 (0.9)	<.001
Blood pressure, mean (SD), mm Hg				
First systolic	157.5 (21.3)	158.7 (20.9)	157.0 (21.5)	<.001
Second systolic	152.0 (20.7)	153.5 (20.2)	151.3 (20.9)	<.001
First diastolic	97.0 (14.0)	96.8 (14.1)	97.1 (13.9)	.37
Second diastolic	94.8 (13.7)	94.6 (13.8)	94.9 (13.7)	.26
Heart rate, mean (SD), beats per min	83.6 (15.7)	82.0 (13.7)	84.3 (16.4)	<.001
Walk-in treatment rate, %^b^	52.0	52.6	51.8	.47

Abbreviations: BMI, body mass index (calculated as weight in kilograms divided by height in meters squared); NA, not applicable; NE, not able to be estimated.

^a^ Data are presented as number (percentage) of patients unless otherwise indicated.

^b^ The walk-in treatment rate was considered the rate of receiving hypertension treatment at the start of the baseline visit.

Table 2 outlines the unadjusted baseline 37-day retention rates between the registration visit and the first follow-up visit overall and by sex and area council. The mean unadjusted 37-day retention rate was 36% (95% CI, 35%-37%) and was similar between males and females. In the Bwari area, females had a higher retention rate compared with males (33% [95% CI, 30%-36%] vs 26% [95% CI, 22%-30%]; *P* = .01), but rates were similar between males and females in other area councils.

**Table 2.** Unadjusted Baseline 37-Day Retention Rates Between the Registration Visit and the First Follow-up Visit Overall and by Sex and Area Council

Area council	Total	37-d Retention rate (95% CI)^a^		
		Males	Females	*P* value
Abaji	0.39 (0.37-0.42)	0.41 (0.37-0.45)	0.39 (0.36-0.42)	.45
Abuja municipal area council	0.30 (0.28-0.31)	0.28 (0.25-0.31)	0.30 (0.28-0.32)	.32
Bwari	0.31 (0.28-0.33)	0.26 (0.22-0.30)	0.33 (0.30-0.36)	.01
Gwagwalada	0.38 (0.36-0.40)	0.37 (0.33-0.41)	0.39 (0.36-0.41)	.44
Kuje	0.52 (0.48-0.55)	0.54 (0.48-0.60)	0.51 (0.46-0.55)	.38
Kwali	0.38 (0.35-0.41)	0.36 (0.32-0.41)	0.39 (0.35-0.43)	.48
Total	0.36 (0.35-0.37)	0.35 (0.34-0.37)	0.36 (0.35-0.37)	.52

^a^ Retention was defined as a follow-up visit to the primary health center within 37 days of the patient’s first (ie, registration) visit.

**Figure 1.**
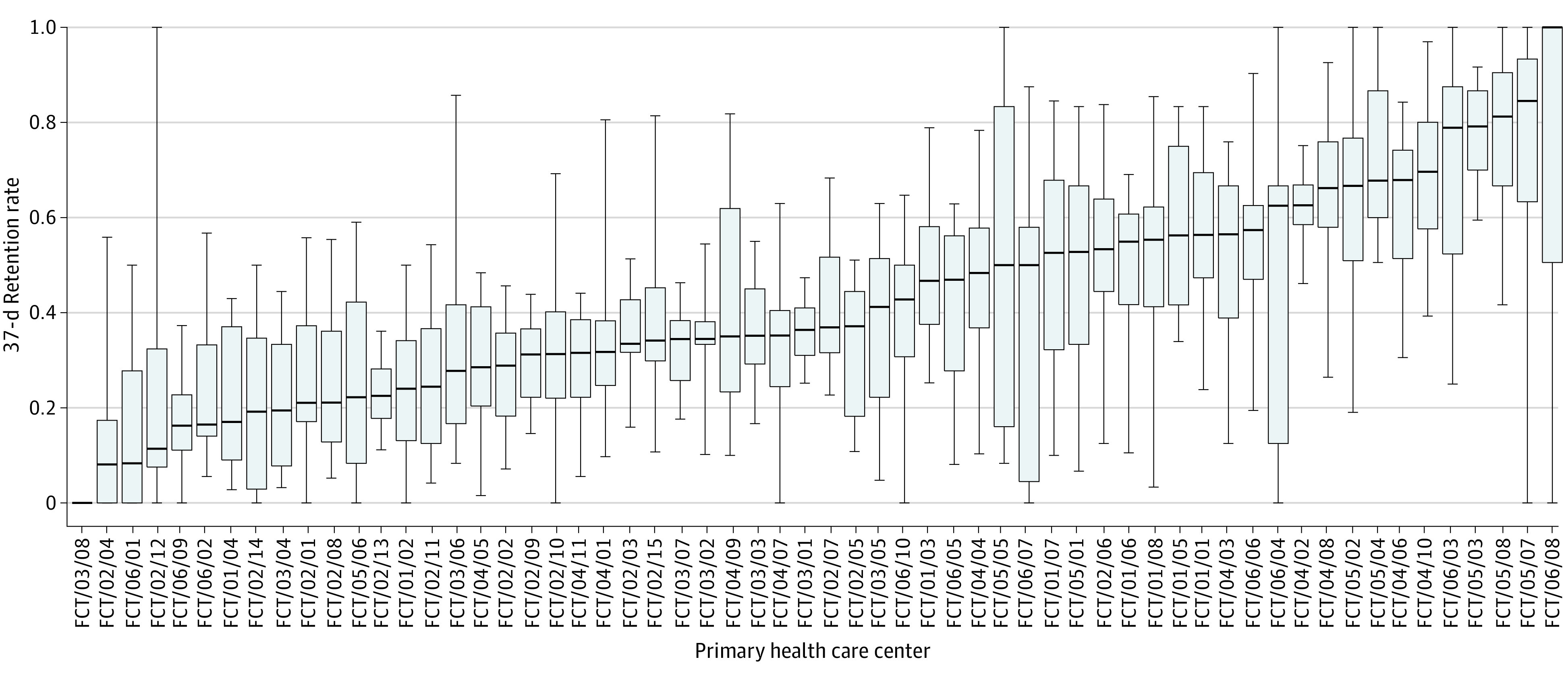
Three-Month Rolling Average 37-Day Retention Rate Between the Registration Visit and First Follow-up Visit for Patients With Hypertension in Primary Health Care Centers in the Federal Capital Territory of Nigeria Inner horizontal lines of the boxes indicate medians; outer horizontal lines of the boxes, interquartile ranges; and whiskers, 95% CIs. The FCT numbers are the study identification numbers for each of the primary health care centers.

The 3-month rolling average 37-day retention rate between the registration visit and the first follow-up visit for patients with hypertension in PHCs in the Federal Capital Territory of Nigeria was 41% (95% CI, 37%-46%), with wide variability among sites (retention rate intraclass correlation coefficient, 0.24) (Figure 1).

eTable 2 in the Supplement shows the characteristics of retention patterns in hypertension care by area council. Among all eligible patients, 35.7% returned to PHCs within 37 days, 14.6% returned between 37 and 90 days, 5.3% returned between 91 and 180 days, 2.7% returned between 181 and 365 days, and 0.6% returned after 365 days. The median time elapsed between the registration visit and the first follow-up visit for all the patients was 39 days (IQR, 32-62 days). The median time elapsed between the first and second visit for those who did not return within 37 days was 74 days (IQR, 53-125 days).

eTable 3 in the Supplement presents the characteristics of staff by area council. The total number of staff was 1007, including 549 full-time and 458 part-time staff. The median number of staff per PHC was 12 (IQR, 7-23), the median number of registered patients per PHC was 177.5 (IQR, 109.0-223.5), and the median number of patients per staff member was 10.5 (IQR 7.0-20.0).

**Figure 2.**
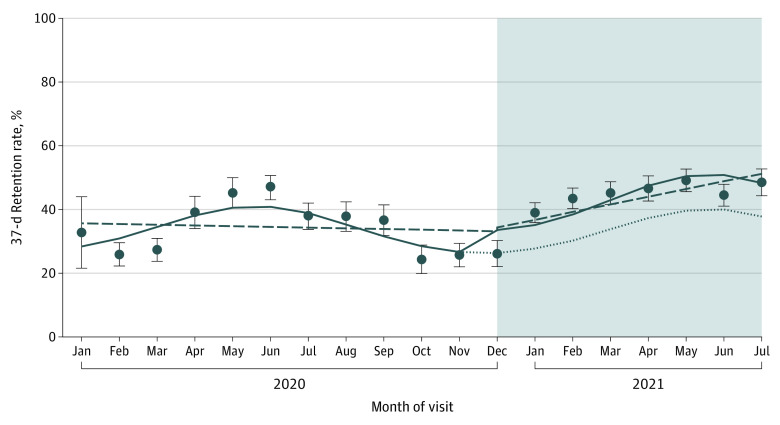
Interrupted Time Series With Level Change Regression Model Adjusted for Seasonality Monthly point estimates are based on the observed retention rate, determined from the date of the first (ie, registration) clinic visit. Circles indicate the mean 37-day retention rate in each month; whiskers, 95% CIs; solid line, predicted 37-day retention rate trend based on the seasonally adjusted regression model; dotted line, predicted 37-day retention rate trend without intervention; dashed line, deseasonalized trend; and shaded area, implementation phase.

Figure 2 shows the interrupted time series regression model with level change adjusted for seasonality. The adjusted root-mean-square error of the model was 0.04. Although the 37-day retention rate modestly decreased overall from January to December 2020, retention increased after the implementation of the WHO HEARTS package from December 2020 (25.6%; 95% CI, 22.1%-30.2%) to July 2021 (48.5%; 95% CI, 44.3%-52.7%) (*P* < .001). The nonlinear trend revealed a potential pattern of seasonality, with higher retention rates after baseline visits in the second quarter of the calendar year compared with other quarters.

Table 3 shows the logistic regression results. The 37-day retention rate was higher among patients who were older (adjusted odds ratio [aOR], 1.01 per year; 95% CI, 1.01-1.02 per year), were female (aOR, 1.11; 95% CI, 1.01-1.23), had a higher body mass index (aOR, 1.01; 95% CI, 1.00-1.02), were treated in the Kuje area council compared with the Abaji area council (aOR, 2.25; 95% CI, 1.25-4.04), received blood pressure–lowering treatment at the registration visit (aOR, 1.27; 95% CI 1.07-1.50), and registered during the postintervention period (aOR, 1.16; 95% CI, 1.06-1.26). Patients who received a new diagnosis of hypertension at their registration were less likely to return within 37 days (aOR, 0.82; 95% CI, 0.74-0.90). The number of staff and number of patients per staff member were not associated with the 37-day retention rate.

**Table 3.** Mixed-Effects, Multivariable Logistic Regression Models to Evaluate Factors Associated With a 37-Day Retention Rate Between the Registration Visit and the First Follow-up Visit

Variable	Model 1, unadjusted OR (95% CI)	Adjusted OR (95% CI)	
		Model 2^a^	Model 3^b^
Age, per year	1.01 (1.01-1.02)	1.01 (1.01-1.02)	1.01 (1.01-1.02)
Sex			
Female	1.03 (0.94-1.12)	1.10 (1.01-1.20)	1.11 (1.01-1.23)
Male	1 [Reference]	1 [Reference]	1 [Reference]
BMI, per unit	1.01 (1.00-1.01)	1.01 (1.00-1.01)	1.01 (1.00-1.02)
Hypertension			
Preexisting diagnosis	1 [Reference]	1 [Reference]	1 [Reference]
New diagnosis at registration	0.76 (0.71-0.83)	0.80 (0.74-0.87)	0.82 (0.74-0.90)
History of diabetes			
No	1 [Reference]	1 [Reference]	1 [Reference]
Yes	1.12 (0.94-1.33)	1.01 (0.85-1.21)	1.01 (0.83-1.22)
Alcohol use			
Noncurrent	1 [Reference]	1 [Reference]	1 [Reference]
Current	1.01 (0.81-1.24)	1.03 (0.83-1.28)	0.86 (0.68-1.08)
Use of fixed-dose combination therapy^c^			
No	1 [Reference]	1 [Reference]	1 [Reference]
Yes	0.98 (0.89-1.08)	0.95 (0.86-1.04)	1.05 (0.94-1.17)
Educational level^d^			
Never attended school	1 [Reference]	1 [Reference]	1 [Reference]
Primary school	0.82 (0.73-0.92)	0.87 (0.77-0.98)	0.99 (0.87-1.13)
Secondary school	0.67 (0.60-0.75)	0.76 (0.68-0.86)	0.96 (0.84-1.10)
High school or more	0.77 (0.70-0.86)	0.86 (0.77-0.96)	0.99 (0.87-1.12)
Area council			
Abaji	1 [Reference]	1 [Reference]	1 [Reference]
Abuja municipal area council	0.65 (0.58-0.73)	0.70 (0.62-0.79)	0.71 (0.43-1.17)
Bwari	0.68 (0.58-0.78)	0.71 (0.62-0.83)	0.59 (0.33-1.05)
Gwagwalada	0.95 (0.84-1.09)	0.99 (0.87-1.13)	1.22 (0.72-2.08)
Kuje	1.66 (1.40-1.96)	1.71 (1.45-2.03)	2.25 (1.25-4.04)
Kwali	0.95 (0.82-1.11)	1.00 (0.86-1.17)	1.05 (0.60-1.83)
BP treatment at registration visit^e^			
No	1 [Reference]	1 [Reference]	1 [Reference]
Yes	1.39 (1.19-1.63)	1.36 (1.17-1.59)	1.27 (1.07-1.50)
BP control at registration visit			
No	1 [Reference]	1 [Reference]	1 [Reference]
Yes	1.05 (0.93-1.18)	0.99 (0.88-1.12)	0.98 (0.86-1.12)
Staff members, including part-time	0.99 (0.99-1.00)	0.99 (0.99-1.00)	0.99 (0.98-1.00)
Patients per staff member	1.00 (0.99-1.00)	1.00 (0.99-1.00)	0.99 (0.98-1.01)
Postintervention period registration	1.13 (1.04-1.23)	1.11 (1.02-1.21)	1.16 (1.06-1.26)
Staff type			
Generalist MDs	0.88 (0.86-0.90)	0.89 (0.86-0.91)	0.89 (0.79-0.99)
Specialist MDs	0.97 (0.94-0.99)	0.96 (0.94-0.99)	0.95 (0.82-1.10)
Nonphysician clinicians or paramedical professionals	1.42 (1.23-1.63)	1.39 (1.20-1.60)	1.34 (0.72-2.51)
Nursing professionals	1.00 (0.99-1.01)	1.00 (0.99-1.01)	0.99 (0.94-1.04)
Pharmacists	0.79 (0.74-0.85)	0.79 (0.73-0.84)	0.81 (0.57-1.15)
Laboratory technicians	0.97 (0.96-0.98)	0.97 (0.96-0.98)	0.97 (0.92-1.02)
Community health extension workers	0.98 (0.97-0.99)	0.98 (0.97-0.99)	0.98 (0.95-1.02)
Junior community health extension workers	0.99 (0.98-0.99)	0.99 (0.98-0.99)	0.98 (0.94-1.01)
Community volunteers	0.99 (0.98-0.99)	0.99 (0.98-0.99)	0.98 (0.96-1.01)
Community health officers	0.82 (0.78-0.85)	0.82 (0.79-0.86)	0.85 (0.71-1.03)
Other^f^	1.00 (1.00-1.01)	1.00 (1.00-1.01)	1.00 (0.98-1.03)

Abbreviations: BMI, body mass index (calculated as weight in kilograms divided by height in meters squared); BP, blood pressure; OR, odds ratio.

^a^ Adjusted for age and sex.

^b^ Adjusted for age, sex, BMI, alcohol use, fixed-dose combination therapy, educational level, and primary health care center (random effect).

^c^ Fixed-dose combination therapy at the start of the baseline visit (ie, walk-in period).

^d^ *P* < .001 for trend across levels of education for 37-day retention rate.

^e^ Defined as patients who received treatment when they completed the clinical visit, including patients who continued treatment or were newly treated.

^f^ Other staff include health attendants, medical record officers, security workers, and cleaners.

## Discussion

### Summary of Results

This study evaluated retention in hypertension care and was embedded in the HTN Program, which is the largest facility-based hypertension program of care in Nigeria and in Africa. The 3-month rolling average retention rate for the registration visit and the first follow-up visit was 41% across 60 public PHCs in the Federal Capital Territory, and wide variability among PHCs was observed. The median duration between the registration visit and the first follow-up visit for all the patients was 39 days but was approximately 2.5 months (74 days) among those who did not return within 37 days. Patients had low levels of education and low rates of smoking and comorbid diseases. Patients who were older, were female, and/or had a higher body mass index were more likely to have a higher retention rate. In addition, receiving blood pressure–lowering treatment during the first visit was also associated with a higher retention rate, suggesting the importance of initial treatment and an association of initial treatment with follow-up in the cascade of hypertension care.

The pattern of short-term retention showed seasonal differences, with higher retention rates after baseline visits in the second quarter of the calendar year compared with other quarters. Furthermore, although the 37-day retention rate modestly decreased in 2020, likely owing to the COVID-19 pandemic, retention increased after the implementation of the WHO HEARTS package starting in December 2020, which included the provision of free blood pressure–lowering medications to patients in January 2021. Although it is not feasible to directly infer a causal relationship, these results suggest that implementation of the WHO HEARTS package is associated with improved retention in hypertension care. Future research is needed to explore the findings observed in the interrupted time series pattern.

Retention rates also differed among and within the 6 area councils, including between sexes and among PHC staffing levels. For example, in Bwari, females had a significantly higher retention rate compared with males, which might be attributable to the geographic and sociodemographic differences in this area. Furthermore, Kuje had the highest retention rate; however, the median number of staff members per PHC in this area was 8, which was lower than the median of 12 staff members per PHC across all areas. Possible reasons for the higher retention rate include the following: (1) Kuje’s staff commitment may have been higher than that in the other areas; (2) Kuje had a stricter quarantine policy after the COVID-19 pandemic than did the other area councils, and patients may have received care only within their community, which may have increased local primary health care visit frequency and thus retention; and (3) most health care workers lived in the same community where the PHCs were located, were already familiar with the patients, and often reminded the patients about follow-up visits. However, there may be a threshold below which fewer staff members would be associated with reduced retention. Patients who registered after the intervention period had a higher retention rate; this finding suggests that the HTN Program was associated with improved short-term retention.

### Results in Context

These results revealed potentially modifiable and nonmodifiable patient, site, area council, and temporal factors that may be associated with differences in retention. Potentially modifiable factors include the provision of free hypertension treatment and the implementation of the WHO HEARTS technical package. Other potentially modifiable factors that were not measured, such as distance to the PHC and operating hours, might be associated with retention. Health systems in low- and middle-income countries also have lower universal health coverage rates for noncommunicable disease care compared with coverage for maternal, neonatal, nutritional, and communicable diseases; this lack of coverage may further limit retention in hypertension care.^26^

The low level of short-term retention reported in the current study may be expected. Studies in low- and middle-income countries have shown even lower levels of follow-up or adherence to clinic appointments. For example, a 2018 study in Kenya reported that only 21% of patients with hypertension had follow-up visits within 6 months, and 63% of those patients were adherent to clinical appointments after the follow-up visits.^27^ A study in Malawi showed that 47% of patients with hypertension had at least 1 follow-up visit within 24 months.^28^ Similar to our findings, that study found retention in care to be higher among older and female patients. The Community-based Hypertension Improvement Project cohort study in Ghana reported that 41% of patients with hypertension had follow-up visits at 6 months, and 25% continued to retain hypertension care at 12 months.^29^ Studies that used longer time horizons (eg, 6 or 12 months) may have reported higher retention rates, but longer time horizons often do not match clinical care models wherein patients are provided 1-month supplies of blood pressure–lowering medications.

Patient retention may be limited by patients’ circumstantial, geographic, and financial access to health care, the perception of the disease processes and patients’ care needs, and external factors such as the COVID-19 pandemic.^11^ Losing patients to follow-up at the retention step of the hypertension care cascade negates the work of all other previous steps, including accurate diagnosis and treatment.^30^ Low availability and cost of medications may deter patients from returning to clinics.^31^ Retention rates in this study improved after HEARTS implementation, which included the provision of free medications; however, retention rates remained suboptimal. Treatment-seeking and management behavior may be further associated with social and religious constructs, such as gender inequity in treatment decision-making, lack of awareness of causes and treatment models, and reliance on herbal and traditional remedies.^32^

Interventions to improve retention have been tested with generally modest effect sizes, but they typically operate only at a single level (eg, patient, practitioner, or facility). For example, behavioral communication-based hypertension educational tools have been shown to be associated with an increase in the retention rate of 9% at 6 months.^33^ Furthermore, a study in Kenya showed that integrated and colocated chronic disease care was associated with an increase in the retention rate of 10.5% over 6 months,^34^ and mobile health messaging increased the retention rate by 10%.^35^ These findings suggest that multilevel interventions such as the WHO HEARTS package may be effective and can potentially address circumstances in which patients’ needs change over time and risk factors, diseases, and health resources are in a continuous state of interaction and flux congruent with complexity models.

Despite the importance of retention in the hypertension care cascade, it is uncertain which strategies are most effective in improving retention and in which contexts. Addressing practitioner-patient communication, financial barriers, and education has been shown to be effective in improving retention.^36,37^ A project that includes a systematic review of interventions to improve retention is under way,^38^ as well as a community participatory research study that will incorporate stakeholder perspectives in Nigeria (ie, patients, community health extension workers, nurses, pharmacists, researchers, administrators, policy makers, and physicians).

### Strengths and Limitations

This study has several strengths. The study collected high-quality data from PHC settings in Nigeria that had not previously conducted hypertension research and included data during the COVID-19 pandemic. The study also included longitudinal follow-up of a large number of patients from a large number of sites at the primary health care level, where most care in Nigeria is delivered. In addition, the study addressed retention, which is an essential but understudied aspect of longitudinal, high-quality hypertension care.

This study also has limitations. First, although the PHCs were sampled using a multistage probability approach to represent care in the Federal Capital Territory, the results may not be generalizable to other states in Nigeria or countries outside Nigeria. Second, although the trained study staff were instructed to use the audit and feedback reports to optimize patient retention, the fidelity of the report used may have been lower in some PHCs than in others. Nevertheless, the study team performed quarterly site-level monitoring and supportive supervision visits to assist in the facilitation activities, including training and education, with real-time feedback to address the barriers to the study procedures, including review of the audit and feedback reports. Third, despite efforts to optimize data quality, including biweekly data status and quality reporting, there were some missing or low-quality data that needed to be excluded. However, the proportion of patients excluded was small (3.5%) and thus not likely to influence the validity of the results. Fourth, we were limited in our ability to understand how the COVID-19 pandemic may have influenced the retention rate, which was beyond the scope of this study. The Delta wave was observed in Nigeria between early December 2020 and early March 2021 (the peak was late January 2021). Although there is some overlap with this wave and the nadir of retention in the current study (October to December 2020), lower retention was observed before the Delta variant was widely observed. However, some patients may have been ill with COVID-19 before the observed Delta wave based on confirmatory test data. Future research planned by the study team includes the qualitative exploration of factors, such as the COVID-19 pandemic, that may be associated with retention among stakeholders, including patients. Future research is needed to explore the findings observed in the interrupted time series pattern.

## Conclusions

The findings of this study suggest that short-term retention in hypertension care is suboptimal and widely variable across the PHCs in the Federal Capital Territory of Nigeria. We identified potentially modifiable and nonmodifiable factors associated with retention. Because of the burden of hypertension, evidence-based strategies for improving retention in hypertension care at the primary health care level are needed to improve hypertension management in Nigeria and likely in other settings.

## Notes

Supplement 1. **eTable 1.** Number of Patients Registered and Number of Patient Visits by Calendar Month and Area Council **eTable 2.** Characteristics of Retention in Hypertension Care by Area Council **eFigure 1.** Example of the Audit and Feedback Report for the Hypertension Treatment in Nigeria Program **eFigure 2.** Flow Diagram of Study Participants **eTable 3.** Characteristics of Primary Health Care Center Staff by Area Council

Supplement 2.
Hypertension Treatment in Nigeria Program Investigators
