# Supplementary material for: Characteristics and Patterns of Retention in Hypertension Care in Primary Care Settings From the Hypertension Treatment in Nigeria Program
Source: JAMA Netw Open. 2022 Sep 6;5(9):e2230025. doi: 10.1001/jamanetworkopen.2022.30025 (PMC9449788; doi:10.1001/jamanetworkopen.2022.30025)
Supplement: Supplement 1. — eTable 1. Number of Patients Registered and Number of Patient Visits by Calendar Month and Area Council eTable 2. Characteristics of Retention in Hypertension Care by Area Council eFigure 1. Example of the Audit and Feedback Report for the Hypertension Treatment in Nigeria Program eFigure 2. Flow Diagram of Study Participants eTable 3. Characteristics of Primary Health Care Center Staff by Area Council [file jamanetwopen-e2230025-s001.pdf]

Supplementary Online Content

Ye J, Orji IA, Baldridge AS, et al; Hypertension Treatment in Nigeria Program Investigators. Characteristics and patterns of retention in hypertension care in primary care settings from the Hypertension Treatment in Nigeria Program. *JAMA Netw Open*. 2022;5(9):e2230025. doi:10.1001/jamanetworkopen.2022.30025

eTable 1. Number of Patients Registered and Number of Patient Visits by Calendar Month and Area Council
eTable 2. Characteristics of Retention in Hypertension Care by Area Council
eFigure 1. Example of the Audit and Feedback Report for the Hypertension Treatment in Nigeria Program
eFigure 2. Flow Diagram of Study Participants
eTable 3. Characteristics of Primary Health Care Center Staff by Area Council

This supplementary material has been provided by the authors to give readers additional information about their work.

© 2022 Ye J et al. *JAMA Network Open*.

eTable 1. Number of patients registered and number of patient visits by calendar month and area council.

**Area**	**Abaji**		**Abuja Municipal Area Council**		**Bwari**		**Gwagwalada**		**Kuje**		**Kwali**		**Total**	
Month	Registration	Visit	Registration	Visit	Registration	Visit	Registration	Visit	Registration	Visit	Registration	Visit	Registration	Visit
January	17	19	1	1	0	0	42	42	0	0	4	6	64	68
February	164	168	88	95	78	78	137	164	42	46	51	52	560	603
March	143	197	166	195	76	103	113	167	73	88	36	63	607	813
April	50	129	102	189	66	104	60	186	23	70	57	108	358	786
May	42	180	130	244	62	135	72	271	46	111	54	130	406	1071
June	120	368	183	358	102	190	166	430	39	154	54	146	664	1646
July	63	353	145	353	101	214	113	424	24	150	80	192	526	1686
August	45	302	135	345	65	176	109	502	24	170	56	192	434	1687
September	44	303	132	356	81	174	72	439	19	176	40	190	388	1638
October	50	358	114	303	79	225	69	412	20	174	28	158	360	1630
November	91	340	199	357	60	147	77	404	21	171	95	222	543	1641
December	46	209	184	353	65	158	77	426	25	165	49	187	446	1498
January, 2021	180	592	271	548	69	192	183	599	84	278	154	408	941	2617
February, 2021	117	539	353	663	96	333	184	576	64	282	87	333	901	2726
March, 2021	140	706	285	827	70	323	130	582	64	388	68	373	757	3199
April, 2021	50	580	210	780	99	353	140	649	67	419	50	311	616	3092
May, 2021	115	716	265	895	126	418	143	704	57	432	70	303	776	3468
June, 2021	161	819	230	933	130	494	146	732	45	423	85	453	797	3854
July, 2021	95	653	145	702	92	453	119	670	57	385	34	339	542	3202
**Total**	**1733**	**7531**	**3338**	**8497**	**1517**	**4270**	**2152**	**8379**	**794**	**4082**	**1152**	**4166**	**10686**	**36925**

© 2022 Ye J et al. *JAMA Network Open*.

eTable 2. Characteristics of retention in hypertension care by area council.[a]

Indicator	Area Council						
	Abaji N=1733	AMAC N=3338	Bwari N=1517	Gwagwalada N=2152	Kuje N=794	Kwali N=1152	Total N=10686
Retention Pattern, N (%)							
Patients who returned ≤ 37 days	683 (39.4)	992 (29.7)	463 (30.5)	824 (38.3)	412 (51.9)	440 (38.2)	3814 (35.7)
Patients who returned in 37-90 days	342 (19.7)	395 (11.8)	193 (12.7)	308 (14.3)	114 (14.4)	207 (18.0)	1559 (14.6)
Patients who returned in 90-180 days	134 (7.7)	139 (4.2)	84 (5.5)	111 (5.2)	47 (5.9)	51 (4.4)	566 (5.3)
Patients who returned in 180-365 days	82 (4.7)	89 (2.7)	59 (3.9)	30 (1.4)	10 (1.3)	20 (1.7)	290 (2.7)
Cumulative follow-up in 365 days	1241 (71.6)	1615 (48.4)	799 (52.7)	1273 (59.2)	583 (73.4)	718 (62.3)	6229 (58.3)
Patients who returned in >365 days	16 (0.9)	12 (0.4)	10 (0.7)	16 (0.7)	5 (0.6)	4 (0.3)	63 (0.6)
Time Elapsed Between 1st and 2nd Visit for all the patients, day, median (IQR)	41 (31, 71)	34 (29, 57)	64 (46, 99)	33 (29, 52)	32 (29, 45)	40 (31, 61)	39 (32, 62)
Time Elapsed Between 1st and 2nd Visit for those who did not return in 37 days, day, median (IQR)	71 (53, 136)	79 (53, 151)	97 (67, 168)	67 (48, 107)	63 (46, 93)	68 (54, 104)	74 (53, 125)

Abbreviations: AMAC=Abuja Municipal Area Council; IQR=interquartile range.
[a]Only considered those who had follow-up visits.

© 2022 Ye J et al. *JAMA Network Open*.

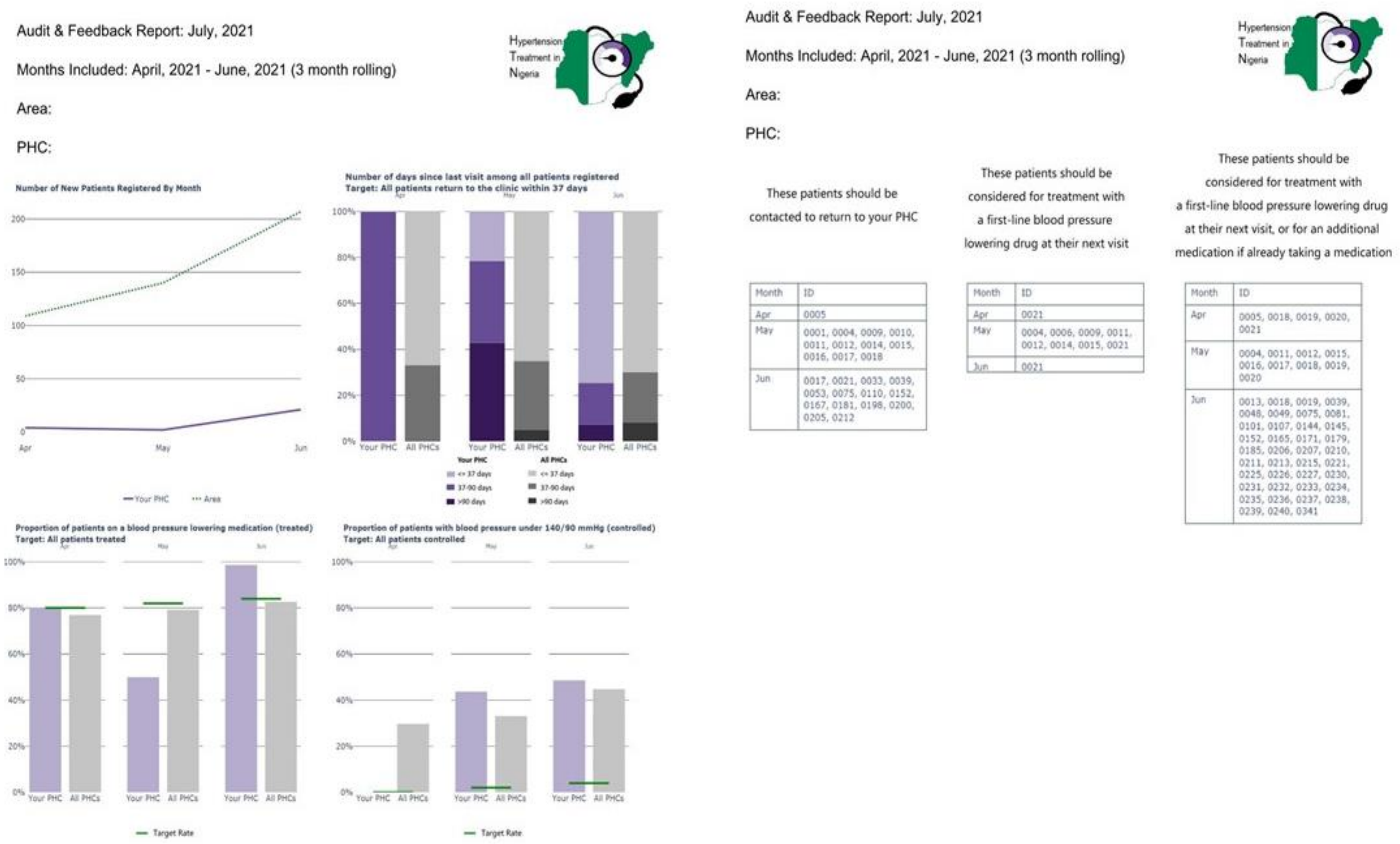

eFigure 1. Example of the audit and feedback report for the Hypertension Treatment in Nigeria Program.

© 2022 Ye J et al. *JAMA Network Open*.

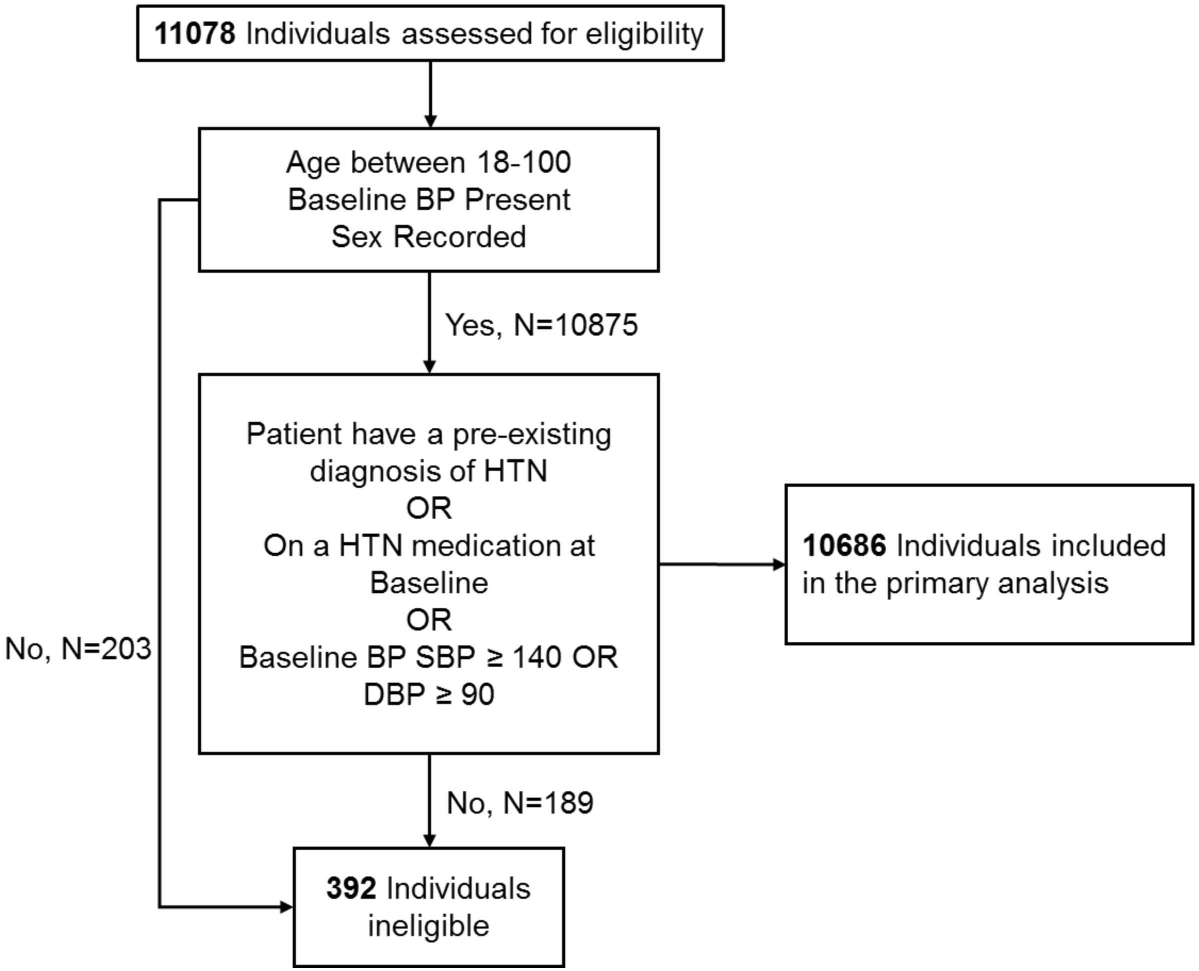

eFigure 2. Flow diagram of study participants.

© 2022 Ye J et al. *JAMA Network Open*.

eTable 3. Characteristics of primary health care center staff by area council.[a]

Area	Abaji	AMAC	Bwari	Gwagwalada	Kuje	Kwali	Total
Number of primary healthcare center (PHCs)	8	15	8	11	8	10	60
Total number of generalist medical doctors	0	22	2	4	2	1	31
Part-time generalist medical doctors	0	21	1	1	1	0	24
Total number of specialist medical doctors	1	0	0	1	0	9	11
Part-time specialist medical doctors	0	0	0	1	0	0	1
Total number of non-physician clinicians/paramedical professionals	0	1	0	0	0	1	2
Part-time non-physician clinicians/paramedical professionals	0	1	0	0	0	0	1
Total number of nursing professionals	9	51	23	33	25	14	155
Part-time nursing professionals	2	25	3	14	11	0	55
Total number of pharmacists	0	1	3	2	0	3	9
Part-time pharmacists	0	0	1	1	0	0	2
Total number of laboratory technicians (medical and pathology)	8	31	13	14	7	9	82
Part-time laboratory technicians (medical and pathology)	1	29	7	9	2	0	48
Total number of community health extension workers	19	65	40	83	38	35	280
Part-time community health extension workers	7	22	15	30	10	3	87
Total number of junior community health extension workers	16	47	20	18	9	15	125

© 2022 Ye J et al. *JAMA Network Open*.

Part-time junior community health extension workers	5	22	14	9	0	2	52
Total number community volunteers	8	55	35	10	1	27	136
Part-time community volunteers	2	43	24	6	0	6	81
Total number community health officers	1	11	5	6	4	7	34
Part-time community health officers	0	5	1	0	0	1	7
Total number of other staff[b]	8	32	26	47	8	21	142
Part-time other staff[b]	3	16	31	35	7	8	100
Total number of staff	70	316	167	218	94	142	1007
Total number of part time staff	20	184	97	106	31	20	458
Number of staff per PHC, median (IQR)	6.5 (6, 10.5)	15 (10, 24)	23.5 (10, 31)	15 (13, 30)	8 (5, 10)	10 (8, 15)	12 (7-23)
Number of part time staff per PHC, median (IQR)	2.5 (1, 4)	7 (5, 20)	9.5 (5.5, 17.5)	12 (2,13)	3.5 (1.5, 6.5)	0 (0, 5)	5.5 (2,12)
Number of registered patients per PHC, median (IQR)	215 (157.5, 239.5)	236 (178, 261)	176.5 (124.5, 198)	180 (121, 204)	66.5 (55, 110.5)	86 (53, 177)	177.5 (109, 223.5)
Number of patient per staff, median (IQR)	25.5 (21.5, 32)	16 (9, 22)	8 (6, 14)	9 (7, 15)	9 (7, 13.5)	7 (5, 10)	10.5 (7, 20)
Number of patient per part-time staff, median (IQR)	72 (52, 111)	22 (12, 53)	15 (10, 29.5)	14 (10, 27)	17 (12, 35)	13.5 (5.5, 39)	20 (12, 52)

Abbreviations: AMAC=Abuja Municipal Area Council; PHC=primary healthcare center.

[a]All the numbers are from the **first** supervision visit at each primary healthcare center (months/year).

[b]Other staff include health attendant, medical record officer, security worker, and cleaner.

© 2022 Ye J et al. *JAMA Network Open*.
