# Supplementary material for: Characteristics and Patterns of Retention in Hypertension Care in Primary Care Settings From the Hypertension Treatment in Nigeria Program
Source: JAMA Netw Open. 2022 Sep 6;5(9):e2230025. doi: 10.1001/jamanetworkopen.2022.30025 (PMC9449788; doi:10.1001/jamanetworkopen.2022.30025)
Supplement: Supplement 2. — Hypertension Treatment in Nigeria Program Investigators [file jamanetwopen-e2230025-s002.pdf]

Supplemental Online Content: Nonauthor Collaborators
*First name, last name, and suffix (if applicable) are required and will appear in PubMed.

*Group Name(s): Hypertension Treatment in Nigeria Program Investigators							
***First Name and Middle Initial(s)**	***Last Name**	***Suffix (eg, Jr, III)**	Academic Degrees	Institution	Location (city, state/province, country)	Role or Contribution, eg, chair, principal investigator	Group (if more than 1 Group listed in the byline) and/or Subgroup (eg, Steering Committee)
Regina	Asuku						
Elizabeth	Ngadda						
Joel	Abu						
Alice	Osuji						
Blessing	Akor						
Charity	Akor						
Christine	Archibong						
Abubakar	Haruna						
Confidence	Joseph-Alo						
Emmanuel	Odo						
Douglas	Okoye						
Grace	Afolabi						
Emmanuel	Okpetu						
Nana	Ripiye						
Dorothy	Ihegazie						
Christian	Ukeh						
Vivian	Chukwuma						
Nicholas	Baamlong						
Sani	Mohammed						
Deborah	Joshua						
Saleh	Ashafa						
Hope	Omeiza						
Mercy	Ikechukwu-Orji						
